# Clinical and Immunological Predictors of Hemorrhagic Fever with Renal Syndrome Outcome during the Early Phase

**DOI:** 10.3390/v14030595

**Published:** 2022-03-14

**Authors:** Geum-Young Lee, Won-Keun Kim, Jin Sun No, Yongjin Yi, Hayne Cho Park, Jaehun Jung, Seungchan Cho, Jingyeong Lee, Seung-Ho Lee, Kyungmin Park, Jongwoo Kim, Jin-Won Song

**Affiliations:** 1 Department of Microbiology, College of Medicine, Korea University, Seoul 02841, Korea; gemyeng002@korea.ac.kr (G.-Y.L.); schanchan@korea.ac.kr (S.C.); yoj0702@korea.ac.kr (J.L.); kmpark0131@korea.ac.kr (K.P.); hotdog442@korea.ac.kr (J.K.); 2 Department of Microbiology, College of Medicine, Hallym University, Chuncheon 24252, Korea; wkkim1061@hallym.ac.kr; 3 Institute of Medical Science, College of Medicine, Hallym University, Chuncheon 24252, Korea; 4 Division of High-Risk Pathogens, Bureau of Infectious Diseases Diagnosis Control, Korea Disease Control and Prevention Agency, Cheongju 28159, Korea; njs2564@gmail.com; 5 Division of Nephrology, Department of Internal Medicine, Dankook University Hospital, Cheonan 31116, Korea; uniqueness@gmail.com; 6 Department of Internal Medicine, Kangnam Sacred Heart Hospital, Seoul 02841, Korea; haynepark798@hallym.or.kr; 7 Department of Preventive Medicine, Gachon University College of Medicine, Incheon 21556, Korea; eastside1st@gmail.com; 8 Chem-Bio Technology Center, Agency for Defense Development, Daejeon 34075, Korea; leeds1104@korea.ac.kr; 9 BK21 Graduate Program, Department of Biomedical Sciences, Korea University College of Medicine, Seoul 02841, Korea

**Keywords:** hemorrhagic fever with renal syndrome, biomarker, early phase, severity, prognosis

## Abstract

The ability to accurately predict the early progression of hemorrhagic fever with renal syndrome (HFRS) is crucial for reducing morbidity and mortality rates in severely affected patients. However, the utility of biomarkers for predicting clinical outcomes remains elusive in HFRS. The aims of the current study were to analyze the serum levels of immune function-related proteins and identify novel biomarkers that may help ascertain clinical outcomes of HFRS. Enzyme-linked immunosorbent assay, Luminex, and bioanalyzer assays were used to quantitatively detect 15 biomarkers in 49 serum samples of 26 patients with HFRS. High hemoglobin (HGB) and low urine output (UO) levels were identified as potential biomarkers associated with the acute HFRS. The serum soluble urokinase plasminogen activator receptor (suPAR) and C-X-C motif chemokine ligand 10 (CXCL10) values increased in the early phase of diseases. Elevated suPAR, interleukin-10 (IL-10), CXCL10, and decreased transforming growth factor-beta 3 (TGF-β3) were representative predictors of the disease severity. Upregulation of the HGB showed a significant correlation with high levels of suPAR and CXCL10. Reduced UO positively correlated with increased suPAR, CXCL10, and TGF-β2, and decreased vascular endothelial growth factor and TGF-β3. The changing HGB and UO criteria, high suPAR, IL-10, CXCL10, and low TGF-β3 of HFRS raise significant awareness for physicians regarding prospective biomarkers for monitoring early warning signs of HFRS. This study provides critical insights into the clinical and immunological biomarkers for disease severity and progression in patients with HFRS to identify early predictions of fatal outcomes.

## 1. Introduction

Hantaviruses are the causative agents of hemorrhagic fever with renal syndrome (HFRS) and hantavirus cardiopulmonary syndrome (HCPS) in humans [1]. Virus transmission usually occurs through the inhalation of aerosols or dust particles contaminated with virus-containing rodent excreta. Hantavirus infection captured worldwide attention during the Korean conflict from 1950 to 1953, during which more than 3000 United Nations troops fell ill with HFRS [2]. In the early 1980s, the causative agent of the disease was reported to be the Hantaan virus (HTNV) isolated from the lungs of a striped field mouse (*Apodemus agrarius*), a natural reservoir [3]. The etiologic agent of nephropathia epidemica, a milder form of HFRS, has been reported to be the Puumala virus (PUUV) found in bank voles (*Myodes glareolus*) in Finland [4]. Pathogenic hantaviruses can cause severe disease in humans, with fatality rates from 1% to 15% [1,5].

HFRS occurs in five distinct phases: febrile, hypotensive, oliguria, polyuria, and convalescence. The febrile phase presents with high fever, malaise, headache, abdominal and lower back pain, nausea, vomiting, hemorrhage, and blurred vision. Hemorrhage manifests as flushing of the face, neck, and thorax, and congestion of the conjunctiva, palate, and pharynx [6]. These signs are followed by the hypotensive phase, which lasts for hours to days and is characterized by thrombocytopenia, which is one of the causes of increased vascular permeability. Acute thrombocytopenia is critical for the diagnosis of HFRS, as it persists throughout orthohantavirus infection. Increased protein loss occurring on days 3–7 of the onset of the febrile phase is a characteristic of severe HFRS and results in massive proteinuria in the majority of cases. In this phase, 15% of patients experience severe clinical shock and mental confusion [1]. Survivors (up to 60%) suffer from acute oliguria, which lasts for 3–7 days. The oliguric stage accounts for approximately one-half of orthohantavirus-related deaths, with fatalities occurring primarily due to renal failure. In the oliguric phase, renal insufficiency leads to an elevation in serum creatinine (sCr), and dialysis is required for approximately 40% of HTNV- and 20% of SEOV-infected patients. During this phase, the tendency to bleed becomes more marked, and various combinations of cerebral hemorrhage, gastrointestinal bleeding, and extensive purpura are observed. Central nervous system symptoms and pulmonary edema occur in severe cases. Surviving patients transition to a diuretic phase, with a diuresis of 3–6 L daily, which lasts for weeks or months. Daily urine volume and duration of the diuretic phase are significantly influenced by the severity of the disease. The convalescent phase takes 2–3 months and is characterized by a progressive recovery of the glomerular filtration rate. Currently, there is no specific antiviral therapy for HFRS. Therefore, early prediction of disease progression has a significant impact on the management of patients with HFRS.

Clinical parameters and immunomodulatory molecule levels in human blood indicate the extent of the inflammatory response to disease severity, which reflects the state and degree of multi-organ dysfunction during the clinical course [7,8,9,10,11,12,13,14,15]. Abnormal human blood levels of pro-inflammatory and anti-inflammatory cytokines, chemokines, and other mediators have been associated with fatal outcomes in HFRS [16,17,18,19,20]. Thrombocytopenia and renal failure are the most common clinical features used as prognostic predictors of severe acute kidney injury (AKI) in patients with HFRS [21,22,23,24]. HFRS-related changes of clinical parameters, including sCr, platelet (PLT), serum albumin (sALB), and white blood count (WBC), have been reported during the acute phase of HFRS [25,26,27]. High serum concentrations of tumor necrosis factor-α (TNF-α), interleukin 6 (IL-6), IL-8, interferon-gamma (IFN-γ), and C-X-C motif chemokine ligand 10 (CXCL10) are found during the febrile, hypotensive, and oliguric phases in severe HFRS cases [28]. Increased vascular endothelial growth factor (VEGF) levels are correlated with the severity of HFRS and degree of kidney damage [29]. Additionally, soluble urokinase-type plasminogen activator receptor (suPAR) level is evaluated as a predictor of severe PUUV infection and as a possible factor involved in the pathogenesis of the disease [30]. The detection of biomarkers that define disease severity and clinical phase is of clinical interest since patients may benefit from rapid diagnosis and systematic treatment. However, studies on early prognostic markers of clinical and laboratory parameters have been limited for fatal outcomes in patients with HFRS.

Here, we enrolled 26 patients with HFRS sampled from the early onset and late phases of symptoms and analyzed the levels of clinical indicators, inflammatory markers, cytokines, and chemokines associated with the early phase and severity of HFRS. The aim of this study was to identify early biomarkers of severity in patients with HFRS. Our study provides early biomarkers that identify the severity of HFRS and predict the subsequent development of severe infections.

## 2. Patients and Methods

### 2.1. Ethics Statement

The human samples were collected under informed consent (AFCH18-IRB-004) with approval for human subjects and case studies from the Korean Armed Forces Capital Hospital.

### 2.2. Clinical Classification of Patients with HFRS

Based on the laboratory tests, the following nine laboratory parameters were reviewed: HGB, PLT, blood urea nitrogen (BUN), sCr, serum protein (sPRT), sALB, UO, WBC, and fever. Moreover, the diagnosis of HFRS was made based on a positive enzyme-linked immunosorbent assay (ELISA) for specific IgG and IgM antibodies to HTNV in the serum.

Based on the clinical classification of HFRS, the patients were classified into three types: (1) mild (n = 1), patients who had kidney injury without oliguria and hypotension; (2) moderate (n = 14), patients who had AKI stage 1 or 2; (3) severe (n = 11), patients who had AKI stage 3, and one or more of the complications with hemodialysis or clinical signs of shock, or blood transfusion. According to the criteria of clinical classification of HFRS disease, the enrolled patients were classified into two groups: (1) early phase (n = 33), the period during the febrile, hypotensive, and oliguric phases, and (2) late phase (n = 16), the period during the diuretic phase. This study included 10 healthy controls.

### 2.3. Quantitative Polymerase Chain Reaction (qPCR) and Reverse Transcription (RT)-PCR

Viral loads were detected in the serum samples using qPCR, which was performed at 95 °C for 10 min, followed by 40 cycles at 95 °C for 15 s, and 60 °C for 1 min using a Power SYBR Green PCR Master Mix (Applied Biosystems, Foster City, CA, USA) on a QuantStudio 5 Real-Time PCR System (Applied Biosystems, Foster City, CA, USA). RT-PCR was performed as previously described using the sequences of the HTNV S segment [31].

Total RNA was isolated from the sera of patient samples using TRI Reagent LS Solution (AMBION Inc., Austin, TX, USA). cDNA was synthesized using a High-Capacity RNA-to-cDNA kit (Applied Biosystems, Foster City, CA, USA) with random hexamers. RT-PCR with primers for detecting the HTNV L, M, and S segments were performed as previously described [32].

### 2.4. Luminex Assay

The concentration of 13 analytes, namely CRP, IFN-γ, IL-1β, IL-6, IL-10, TNF-α, CXCL10, GM-CSF, CXCL9, VEGF-A, TGF-β1, TGF-β2, and TGF-β3, were determined using Luminex assay (Luminex, Austin, TX, USA). Biomarker levels were measured using a MILLIPLEX MAP Human Cytokine/Chemokine Magnetic Bead Panel Kit, Human Cytokine/Chemokine Magnetic Bead Panel III Kit, Human Cardiovascular Disease (CVD) Magnetic Bead Panel 3 Kit, and TGF-β1, 2, and 3 Magnetic Bead Kit on a Luminex 200 analyzer (Luminex, Austin, TX, USA) in duplicate according to the manufacturer’s instructions. The data were analyzed using a 5-parameter logistic or spline curve-fitting method for calculating analyte concentrations in the samples.

### 2.5. ELISA

The suPAR levels were determined using a suPARnostic^®^ AUTO Flex ELISA (ViroGates, Birkerød, Denmark) according to the manufacturer’s instructions. Optical density was measured using a microplate reader (Tecan, Männedorf, Switzerland) at 460 nm.

### 2.6. Cell-Free DNA (cfDNA) Extraction and Quantification

cfDNA was extracted from serum samples of patients with HFRS using the NucleoSpin^®^ Plasma XS Kit (MACHEREY-NAGEL GmbH & Co., Duren, Germany). The amount of cfDNA was measured using an Agilent High Sensitivity DNA Kit and a Bioanalyzer 2100 instrument (Agilent Technologies, Santa Clara, CA, USA) according to the manufacturer’s instructions.

### 2.7. Statistical Analysis

Statistical analysis was performed using GraphPad Prism 7.04 (GraphPad Software, San Diego, CA, USA). Significant differences in the serum concentrations of clinical factors were determined using nonparametric *t*-tests. The nonparametric one-way ANOVA was used to compare the differences of immunological factors among patients with HFRS. Correlations were assessed using a linear regression correlation test with 95% confidence intervals; *p*-values were adjusted for multiple comparisons, and differences were considered significant at *p* < 0.05.

## 3. Results

### 3.1. Patient Characteristics and Clinical Findings

The study population included 26 patients with HFRS infected with HTNV (Table 1). The most common symptoms reported in patients were fever (96%), followed by nausea (31%), headache (19%), and diarrhea (12%). Additional symptoms included myalgia, flank pain, chest pain, sore throat, dizziness, dyspnea, and weakness in 4%. AKI was observed in 96% of the patients. Pathological findings in pulmonary edema and hypotension were observed in 35% and 31% of patients, respectively. Approximately 77% of enrolled patients were admitted at the intensive care unit (ICU) and were patients with HFRS, and treatment with transfusion, mechanical ventilation, and hemodialysis was required in 12%, 4%, and 4%, respectively. Furthermore, the mortality rate was 4% in patients with HTNV infection.

### 3.2. Laboratory Diagnosis of HTNV-Infected HFRS Patients

Of the 26 patients with HFRS, 1 was classified as mild, 14 as moderate, and 11 as severe (Table 2). The time from disease onset to hospitalization was 5–15 days, actual treatment time was 4–17 days, and treatment time for ICU admitted patients was 2–5 days. Among them, one patient died due to a fatal outcome of the disease. One patient (ROKA15-5) with mild HFRS was seronegative for anti-HTNV IgM and IgG antibodies. Of the patients with moderate HFRS, 29% and 86% presented with HTNV-specific IgM and IgG antibodies, respectively. IgM and IgG antibodies in patients with severe HFRS were detected in 9% and 82%, respectively. HTNV RNA was detected only in moderate (79%) and severe (91%) samples.

### 3.3. Clinical and Immunological Biomarkers Associated with Early and Late Phases of HFRS

**Figure 1:**
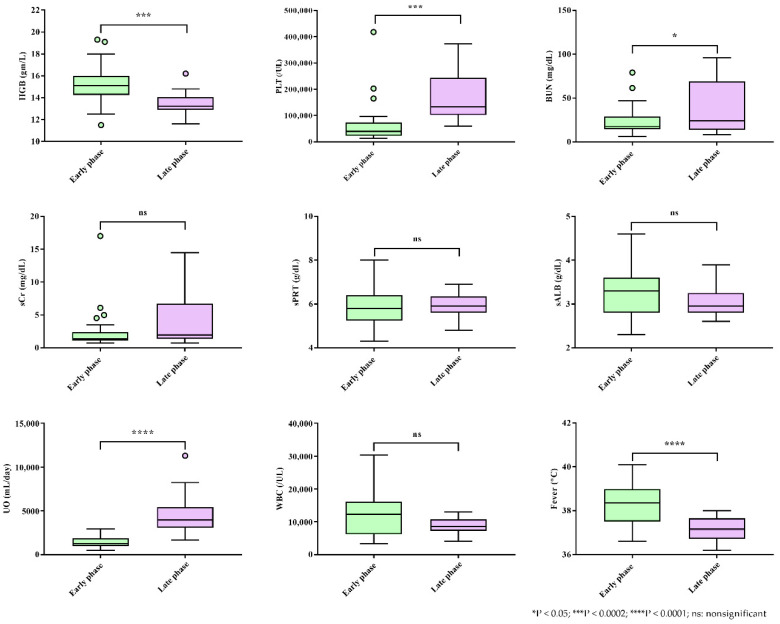
Levels of clinical and laboratory parameters during early and late phases in patients with hemorrhagic fever with renal syndrome (HFRS).

**Figure 2:**
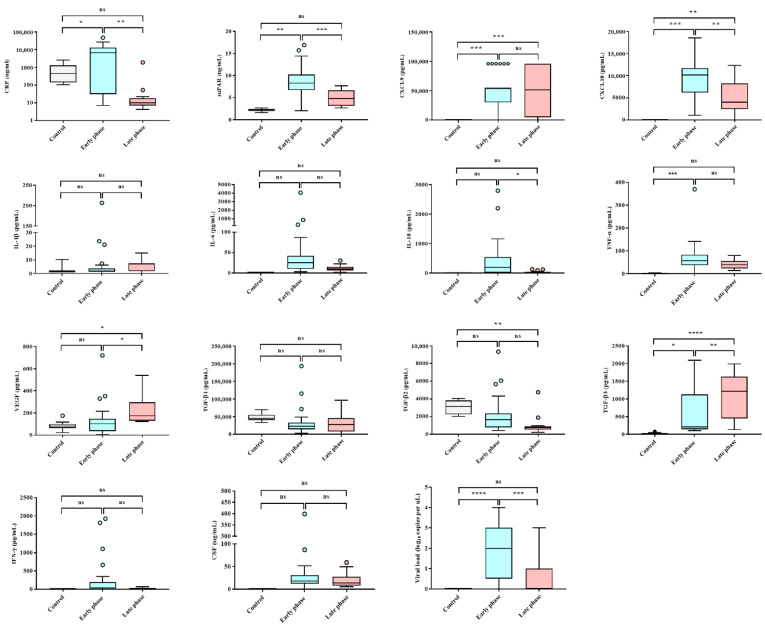
Levels of inflammatory markers, cytokines, and chemokines during early and late phases in patients with hemorrhagic fever with renal syndrome (HFRS). * *p* < 0.05; ** *p* < 0.01; *** *p* < 0.0002; **** *p* < 0.0001.

Patients with acute HFRS presented with fever and high levels of HGB, CRP, suPAR, CXCL9, CXCL10, TGF-β3, TNF-α, and HTNV loads but low levels of PLT, BUN, and UO (Figure 1 and Figure 2). The late phase of the disease was marked by increased VEGF levels and decreased TGF-β2 levels. The laboratory parameters were recorded, including low levels of CRP, suPAR, IL-10, CXCL10, and high levels of TGF-β3, during disease progression. Serum HTNV loads were positively correlated with the levels of HGB, PLT, BUN, sCr, sPRT, UO, suPAR, IL-10, CXCL10, VEGF, and cfDNA, as well as fever (Figure S1). Notably, HGB, UO, suPAR, and CXCL10 were specific markers for patients with acute HFRS.

### 3.4. Clinical and Immunological Biomarkers Associated with HFRS Severity

**Figure 3:**
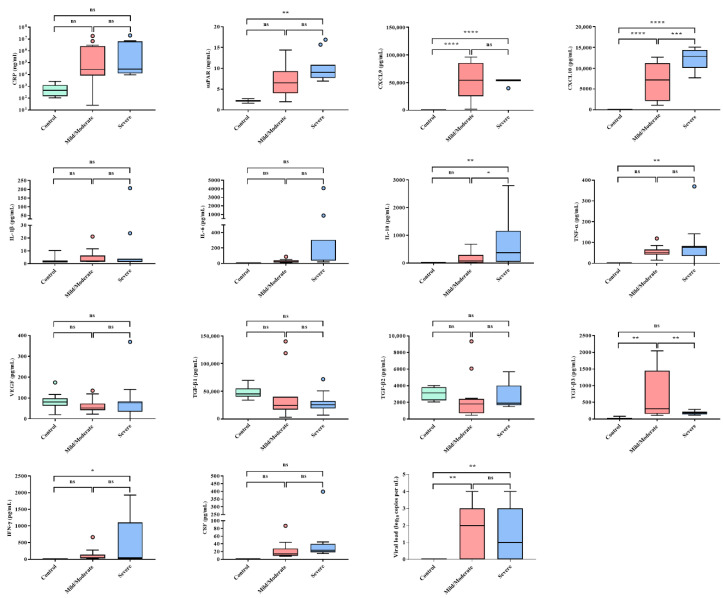
Levels of inflammatory markers, cytokines, and chemokines for disease severity in the patients with hemorrhagic fever with renal syndrome (HFRS). * *p* < 0.05; ** *p* < 0.01; *** *p* < 0.0002; **** *p* < 0.0001.

**Figure 4:**
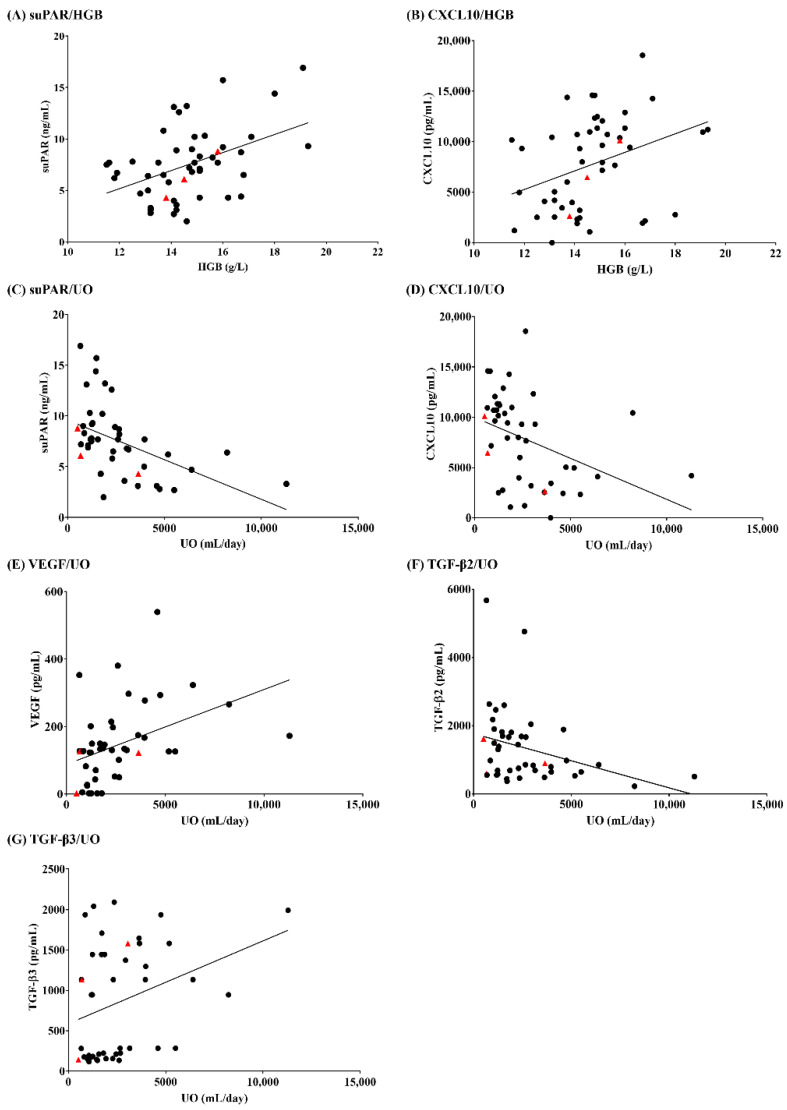
Correlation of hemoglobin (HGB) and urine output (UO) with immunological biomarkers in patients with hemorrhagic fever with renal syndrome (HFRS).

**Figure 5:**
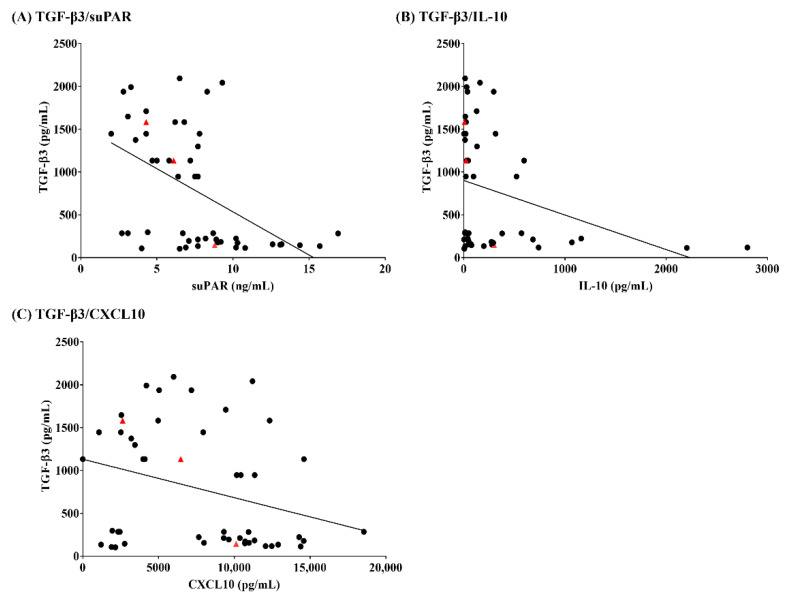
Correlation of TGF-β3 with suPAR, IL-10, and CXCL10 in patients with hemorrhagic fever with renal syndrome.

High levels of CXCL9, CXCL10, and TGF-β3 were observed in patients with mild/moderate HFRS (Figure 3). Meanwhile, patients with severe HFRS exhibited high levels of suPAR, IL-10, CXCL9, CXCL10, TNF-α, and IFN-γ. Severe HFRS cases showed a significant increase in sCr levels, but not in HGB, PLT, BUN, sPRT, sALB, UO, and WBC levels and fever (Figure S2). Correlation analyses demonstrated that the serum levels of suPAR and CXCL10 were upregulated when HGB levels were high (Figure 4). Reduced UO levels were correlated with high levels of serum CXCL10, suPAR, and TGF-β2, while severe HFRS, defined by low UO levels, corresponded to low levels of VEGF and TGF-β3. High levels of suPAR, IL-10, and CXCL10 were associated with low TGF-β3 values, which is likely indicative of fatal outcomes in patients with HFRS (Figure 5).

### 3.5. Quantitative Analysis of Cell-Free DNA (cfDNA) for Patients with HFRS

The presence of plasma cfDNA in patients with HFRS was observed in low-molecular-weight patterns, ranging from 150 bp to 200 bp (Figure S3). In most patients with HFRS, low-molecular-weight bands were visible during the early phase (days 1–6 after onset), but DNA level was markedly weakened in the late phase (days 7–10 after onset). The cfDNA level was 4683 ± 1363 ng/mL during the early phase and 1087 ± 366.1 ng/mL in the late phase. High cfDNA levels were correlated with high HGB, suPAR, IL-10, CXCL10, and TGF-β2 levels (Figure S4).

## 4. Discussion

**Figure 6:**
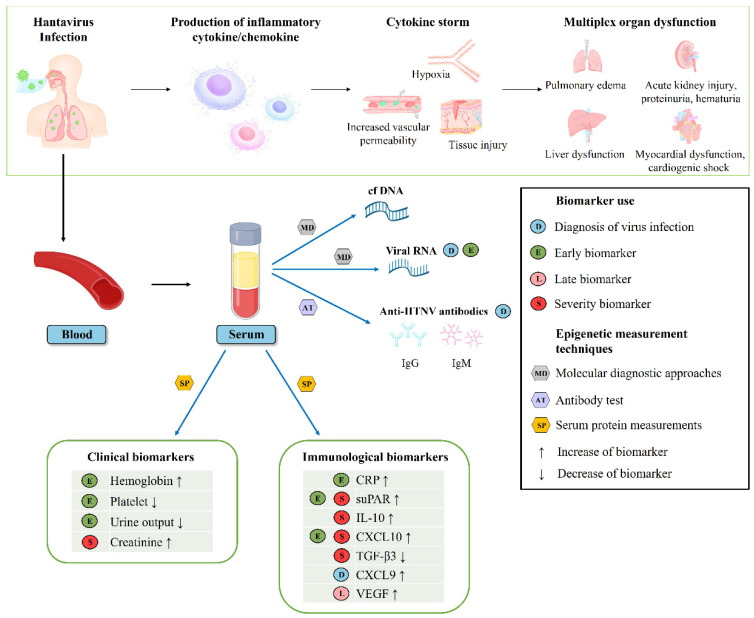
A schematic model summarizing the predictors of clinical progression and outcome in patients with hemorrhagic fever with renal syndrome (HFRS).

This study extends our current knowledge regarding the clinical and immunological biomarkers for the disease phase and severity of HFRS (Figure 6). The use of clinical- and immune-based laboratory biomarkers facilitates a rapid diagnosis for the early identification of patients at risk of severe illness in infectious diseases. Although there are a large number of laboratory parameters routinely tested in patients with HFRS, the evaluation of severity and outcome using laboratory parameters remains challenging owing to the complicated clinical courses and characteristics of HFRS. The aims of this study were to analyze the serum levels of immune function-related proteins, characterize clinical and immunological parameters, and identify novel biomarkers that may help ascertain clinical outcomes in HFRS.

Critical patients with HFRS in the hypotensive and oliguric phases are combined with various fatal complications. They may manifest obvious oliguria with AKI. During this period, timely and systematic diagnosis systems are essential because they have a direct impact on the final prognosis of patients. While extensive efforts have been focused on identifying markers for the diagnosis of kidney injury [33], our current indication of acute renal impairment still relies on the criteria of AKI represented by sCr and UO. However, many studies exclude UO as a criterion when monitoring the clinical severity of patients with AKI due to the technical difficulty of accurate collection and the complexity of interpretation [34]. A previous study reported that increased sCr is a temporary sign associated with the early phase in patients with HFRS [25,26]. However, a recent study showed that sCr criteria alone can miss approximately 20% of patients with AKI when monitoring renal function [35]. Indeed, UO was associated with mortality in patients with AKI in the ICU population [34]. Of the nine routinely tested laboratory parameters, the levels of HGB, PLT, BUN, UO, and fever during the early phase were significantly different (*p* < 0.05) in our study. Furthermore, the patients had significantly lower levels of UO in the early phase (1488 ± 128.7 mL/day) than in the late phase (4622 ± 602.6 mL/day). Our results indicated that patients with severe HFRS had lower levels of UO (991.6 ± 191.1 mL/day). AKI was common in the early phase of HFRS, which may affect the levels of UO to a certain degree. Our data demonstrated that UO is a better indicator of the early phase than sCr. These findings suggest that physicians may significantly improve the diagnostic outcomes for acute HFRS by monitoring UO in combination with other early biomarkers.

In clinical cases, the increased HGB level is a compensatory phenomenon that indicates the degree of hypoxia and vasoconstriction [36,37,38]. A previous study suggested that high serum concentrations may impede uteroplacental circulation during pregnancy, causing placental infarction, growth retardation, and ultimately fetal death [37]. Moreover, high HGB concentrations increase the risk of pregnancy-induced hypertension [36]. A population-based prospective study demonstrated that serum HGB level was significantly associated with the risk of incident nonalcoholic fatty liver disease [39]. A significant association between HGB and atherosclerosis has also been observed in a recent study [40]. However, to the best of our knowledge, no prospective studies have investigated the association between serum HGB levels and HFRS. According to the pathophysiologic mechanism of HFRS, severe plasma leakage, massive bleeding, and profound shock may lead to tissue hypoperfusion and hypoxia, which can result in cardiac, renal, and hepatic injury [41]. On day 26 after returning from Bolivia in 2006, a healthy 15-year-old girl was diagnosed with fatal HCPS after exposure to rodent droppings [42]. The patient eventually died due to pulmonary edema, diffuse alveolar damage, and lymphoid inflammation in the pulmonary interstitium. The patient had repeated HGB levels of 20.6 g/dL prior to cardiac arrest. In this study, the serum HGB (15.73 ± 0.42 g/dL) in the early phase was significantly higher than that in the late phase. Additionally, our results suggest a possible association between HFRS severity and HGB. These observations may be explained by the fact that the HGB values during the early phase are a major predictive factor for prognosis, probably because high levels of HGB may be closely related to severe hypoxia in acute HFRS.

suPAR levels reflect the degree of viral infections in several infectious diseases and cancer [43]. Plasma suPAR levels increased markedly during acute PUUV and Dobrava–Belgrade virus (DOBV) infections and were associated with the severity of HFRS and Crimean–Congo hemorrhagic fever [30,44]. High suPAR concentrations indicate poor outcomes and early mortality in patients with sepsis [9]. Studies in patients with coronavirus disease 2019 (COVID-19) also showed that high suPAR levels were evaluated as a potential early biomarker indicating the need for intensive care admission [12]. This study demonstrated that the levels of suPAR during HTNV infection increased significantly in the early phase. Moreover, suPAR levels were significantly higher in patients with severe HFRS. These results indicate that suPAR may serve as a reliable early biomarker for HTNV infection, which is closely related to the severity of HFRS.

The immunomodulatory cytokine IL-10 plays a critical role in controlling excessive immune responses during infections and autoimmunity, mainly by inhibiting the production of pro-inflammatory cytokines in various cell types. Recent studies have shown that IL-10 predicts the development and clinical course of diseases. High serum values of IL-10 were found in patients with a severe course of dengue hemorrhagic fever and Argentine hemorrhagic fever [45,46]. Notably, elevated IL-10 levels are associated with a poor prognosis of patients with COVID-19 [47]. High serum IL-10 levels were associated with the acute phase in patients with PUUV and DOBV infections [48,49]. Consistent with previous studies, serum IL-10 levels increased significantly in patients with severe HFRS compared with both the mild/moderate and control groups in our study. This study revealed that IL-10 may be a potentially important biomarker indicative of severity in patients with HFRS.

Chemokines are involved in regulating leukocytes during hematopoiesis, immune responses, and inflammation [50]. Currently, ligands of CXCR3 have been intensely investigated for CXCL9, CXCL10, and CXCL11. In an animal model of dengue virus (DENV) infection, reduced recruitment of effector T cells to sites of DENV infection in CXCR3-deficient mice impaired viral clearance and thus increased mortality rates [51]. Additionally, CXCL9 was highly upregulated in patients with Andes virus, PUUV, and DENV infections [52,53,54]. This result revealed that increased CXCL9 was observed in both early and late phases compared to that in healthy controls. Our results showed that upregulation of CXCL9, regardless of disease severity, was characteristic of HFRS throughout the course of the disease after HTNV infection. In addition, CXCL10 expression is responsible for recruiting activated T cells to sites of tissue inflammation. In PUUV infection, serum CXCL10 levels were significantly associated with the early phase of HFRS [10]. CXCL10 showed significantly higher values in severe patients than in mild/moderate patients and healthy controls. Our data suggest that high CXCL10 levels may be a prognostic biomarker indicative of the early phase and disease severity in patients with HTNV infection.

TGF-β is a potent cytokine with diverse effects on both immunosuppressive and anti-inflammatory responses [55]. The TGF-β proteins, TGF-β1, TGF-β2, and TGF-β3, control the initiation and resolution of inflammatory responses through the regulation of chemotaxis and activation of leukocytes in the periphery, including lymphocytes, natural killer cells, dendritic cells, macrophages, mast cells, and granulocytes [56]. Low TGF-β1 and TGF-β2 expression was associated with severe courses of PUUV infection [57]. Low TGF-β1 levels were correlated with high creatinine and low platelet levels, while TGF-β1 levels were increased during the late phase of the disease [58]. Meanwhile, to the best of our knowledge, studies on TGF-β3 in hantaviruses remain unknown. In our study, TGF-β3 levels were lower in patients with severe HFRS than in those with mild/moderate HFRS. We found that severe HFRS cases, characterized by low UO levels, were significantly correlated with low TGF-β3 levels. In addition, low TGF-β3 levels were positively correlated with high suPAR, IL-10, and CXCL10 levels. Thus, these results demonstrate that TGF-β3 is a potential prognostic biomarker for the severity of HFRS.

The plasma cfDNA level in the early phase of HFRS is correlated with HTNV load and disease severity [59]. We also observed that high cfDNA concentrations during HTNV infection were associated with higher viral loads. Intriguingly, high HGB, suPAR, IL-10, and CXCL10 levels were correlated with high cfDNA concentrations. These results support the correlation between these biomarkers and disease severity in HFRS.

This study was limited by the small number of patient samples and the incompleteness of some laboratory records. A large-scale and continuous survey of the potential biomarkers presented in this study should be conducted by further characterizing the clinical progression of HFRS through quantification of serum protein levels of clinical and laboratory parameters. Additionally, further prospective studies are needed to better understand the pathogenesis of biomarkers in HFRS.

Overall, HGB and UO may serve as potential biomarkers for the diagnosis of acute HFRS. Increased suPAR and CXCL10 levels could be early markers of the disease in patients with HFRS. High suPAR, IL-10, and CXCL10, and low TGF-β3 levels can be utilized as prognostic indicators of HFRS severity. Thus, this study provides significant insights into the clinical and immunological biomarkers of the progression and severity of HFRS. Furthermore, the measurement of these biomarkers might improve the clinical evaluation of patients with HFRS by enabling physicians to diagnose fatal outcomes early and predict prognosis.

## Figures and Tables

**Table 1:** Clinical manifestations and outcomes of patients with hemorrhagic fever with renal syndrome.

	n (%)
Symptoms	
Fever	25/26 (96)
Nausea	8/26 (31)
Headache	5/26 (19)
Diarrhea	3/26 (12)
Myalgia	1/26 (4)
Flank pain	1/26 (4)
Chest pain	1/26 (4)
Sore throat	1/26 (4)
Dizziness	1/26 (4)
Dyspnea	1/26 (4)
Weakness	1/26 (4)
**Comorbidities**	
Acute kidney injury	25/26 (96)
Pulmonary edema	9/26 (35)
Hypotension	8/26 (31)
**Treatments and outcomes**	
ICU admission	20/26 (77)
Transfusion	3/26 (12)
Mechanical ventilation	1/26 (4)
Hemodialysis	1/26 (4)
Mortality	1/26 (4)

Abbreviations: ICU, intensive care unit.

**Table 2:** Demographic and clinical characteristics of Hantaan virus (HTNV)-infected hemorrhagic fever with renal syndrome patients.

	Mild (n = 1 ^a^)	Moderate (n = 14)	Severe (n = 11)
Demographics			
Age, years ^a^	23	21–23	21–23
Hospital stay, days ^a^	5	9–11	11–15
Actual treatment, days ^a^	4	10–12	13–17
ICU treatment, days ^a^	2	2–4	3–5
Laboratory tests			
Anti-HTNV IgM positivity	0	4 (29%)	1 (9%)
Anti-HTNV IgG positivity, titers ^b^	0	12 (86%), 1:3904 ± 1598	9 (82%), 1:1844 ± 1458
HTNV RT-PCR positivity	0	11 (79%)	10 (91%)

Abbreviations: ICU = intensive care unit. IgG = immunoglobulin G. IgM = immunoglobulin M. RT-PCR = reverse transcription-polymerase chain reaction. ^a^ ROKA15-5, one of the mild patients, was negative for both anti-HTNV IgG and RT-PCR at the initial phase (collection date: 25 December 2015). Then, the patient was diagnosed with HTNV infection by RT-PCR at the diuretic phase (collection date: 26 December 2015). ^b^ Each parameter is presented as the mean ± standard deviation.

## Data Availability

All the data generated for this publication have been included in the current manuscript.

## Supplementary Materials

The following supporting information can be downloaded at: https://www.mdpi.com/article/10.3390/v14030595/s1, Figure S1: Correlation between biomarkers and viral loads in patients with hemorrhagic fever with renal syndrome; Figure S2: Levels of clinical and laboratory parameters for disease severity in the patients with hemorrhagic fever with renal syndrome (HFRS); Figure S3: Qualitative analysis of cell-free DNA in mild, moderate, and severe patients with hemorrhagic fever with renal syndrome (HFRS); Figure S4: Correlation between biomarkers and cell-free DNA (cfDNA) in patients with hemorrhagic fever with renal syndrome.
