# Supplementary material for: Clinical and Immunological Predictors of Hemorrhagic Fever with Renal Syndrome Outcome during the Early Phase
Source: Viruses. 2022 Mar 14;14(3):595. doi: 10.3390/v14030595 (PMC8954228; doi:10.3390/v14030595)
Supplement: Supplementary file 1 [file viruses-14-00595-s001.zip › viruses-1589207-supplementary.pdf]

Supporting Information

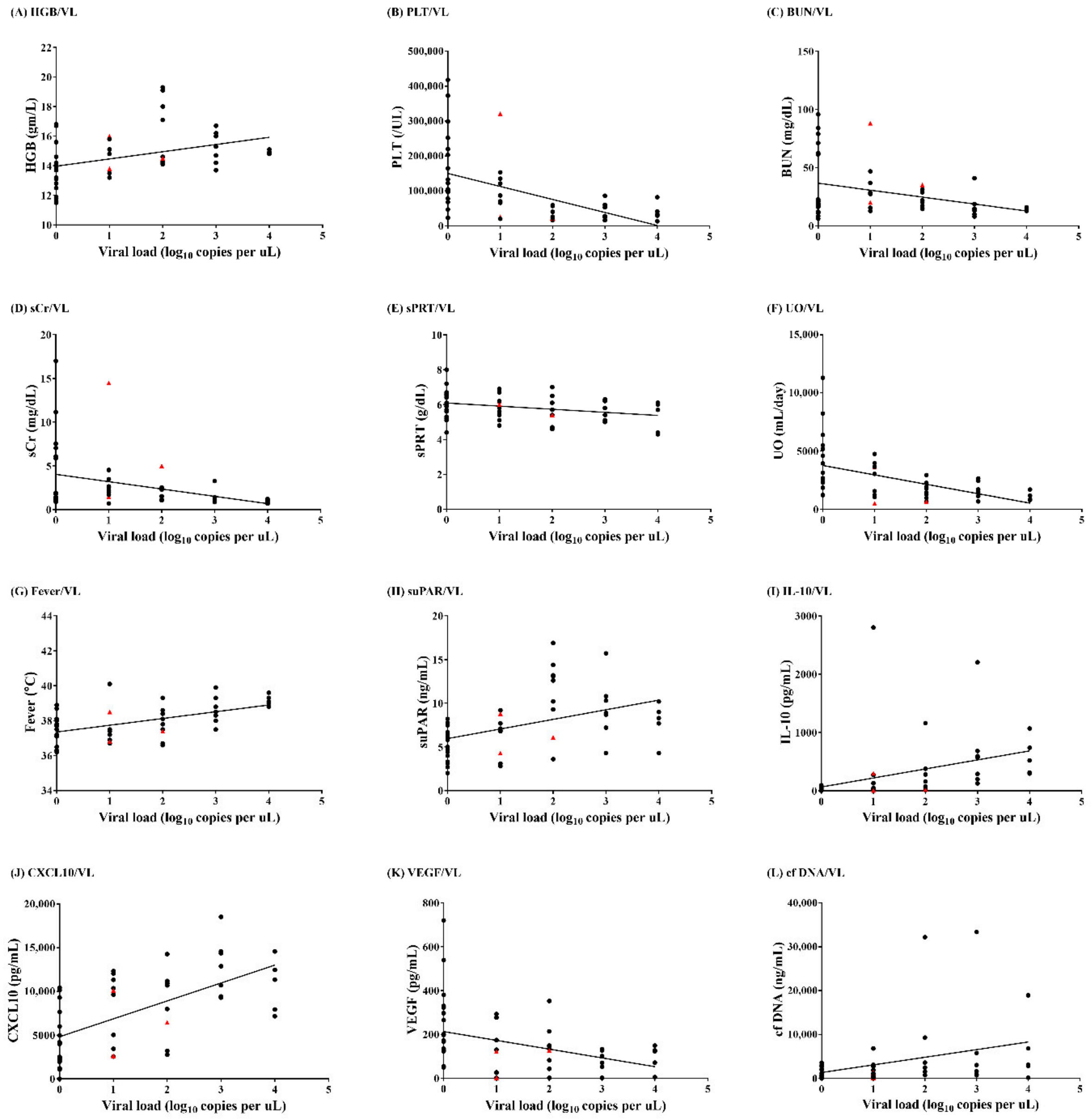

Figure S1. Correlation between biomarkers and viral loads in patients with hemorrhagic fever with renal syndrome. The concentrations of HGB ($P = 0.0054$), PLT ($P < 0.0001$), BUN ($P = 0.0095$), sCr ($P = 0.0168$), sPRT ($P = 0.0257$), UO ($P = 0.0003$), Fever ($P < 0.0001$) correlated with the viral loads of Hantaan virus (HTNV) (A–G). Serum suPAR ($P = 0.0008$), IL-10 ($P = 0.0017$), CXCL10 ($P < 0.0001$), VEGF ($P = 0.0033$), and cfDNA ($P = 0.0215$) showed a correlation with the viral loads (H–L). The correlation coefficient between the parameters is depicted by a straight line. Serum levels from patients who died (n=3) during HTNV infection are shown in red triangles. Black circles indicate the measured 46 serum samples from 26 patients with HFRS. Cell-free DNA, cfDNA; BUN, blood urea nitrogen; HGB, hemoglobin; PLT, platelet; sCr, serum creatinine; sPRT, serum protein; UO, urine output; VL, viral load.

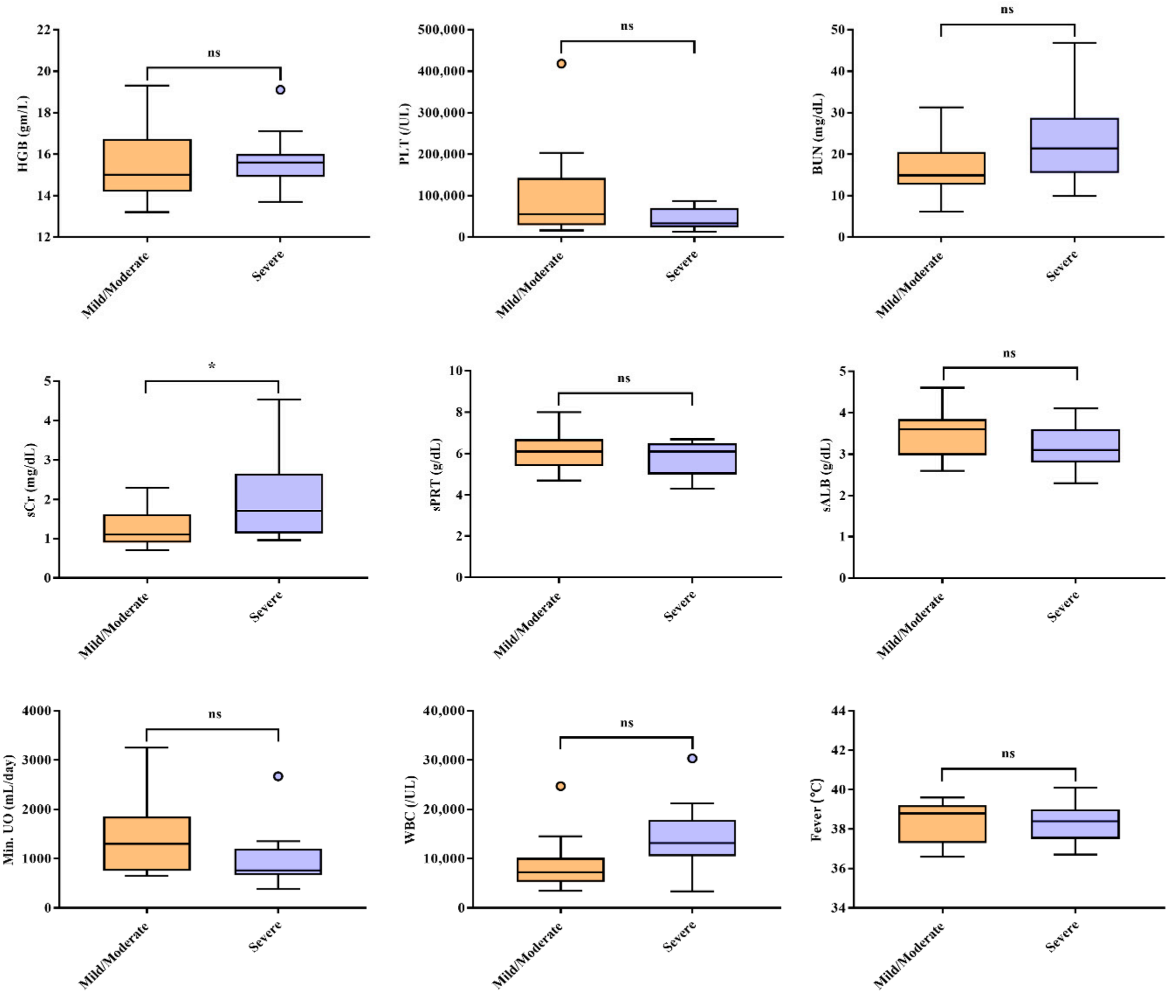

*P < 0.05; ***P < 0.0002; ****P < 0.0001; ns: nonsignificant

Figure S2. Levels of clinical and laboratory parameters for disease severity in patients with hemorrhagic fever with renal syndrome (HFRS). Based on acute kidney injury (AKI) severity, patients with HFRS were classified into two types: mild/moderate disease (AKI stage 1 and 2), and severe disease (AKI stage 3). Box and whiskers were compared using unpaired Student's t-test. Box plots are presented as the mean±standard error. Whiskers represent maximum values excluding outliers, which are represented by dots. Comparison between two groups: *P < 0.05; ns: nonsignificant. Fever information of 2 moderate patients was excluded in this study. BUN, blood urea nitrogen; HGB, hemoglobin; PLT, platelet; sCr, serum creatinine; sPRT, serum protein; sALB, serum albumin; UO, urine output; WBC, white blood counts.

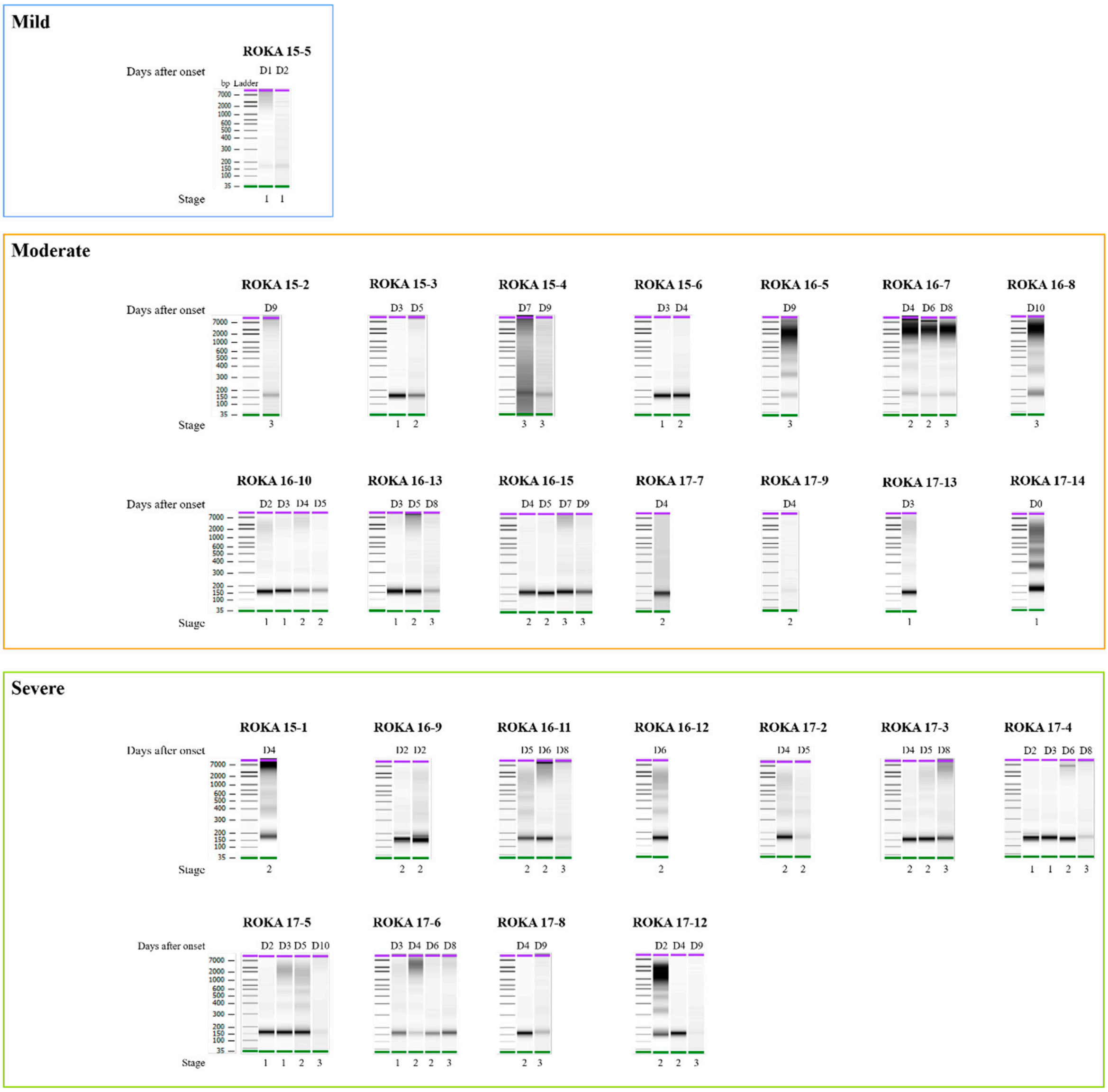

Figure S3. Qualitative analysis of cell-free DNA in mild, moderate, and severe patients with hemorrhagic fever with renal syndrome (HFRS). The low weight (150 bp to 200 bp) solid lines indicate the expected size DNA band. All patients with HFRS were classified into three stages: 1) hypotensive phase (days 1–3 after onset); 2) oliguric phase (days 2–6 after onset); and 3) diuretic phase (days 7–10 after onset).

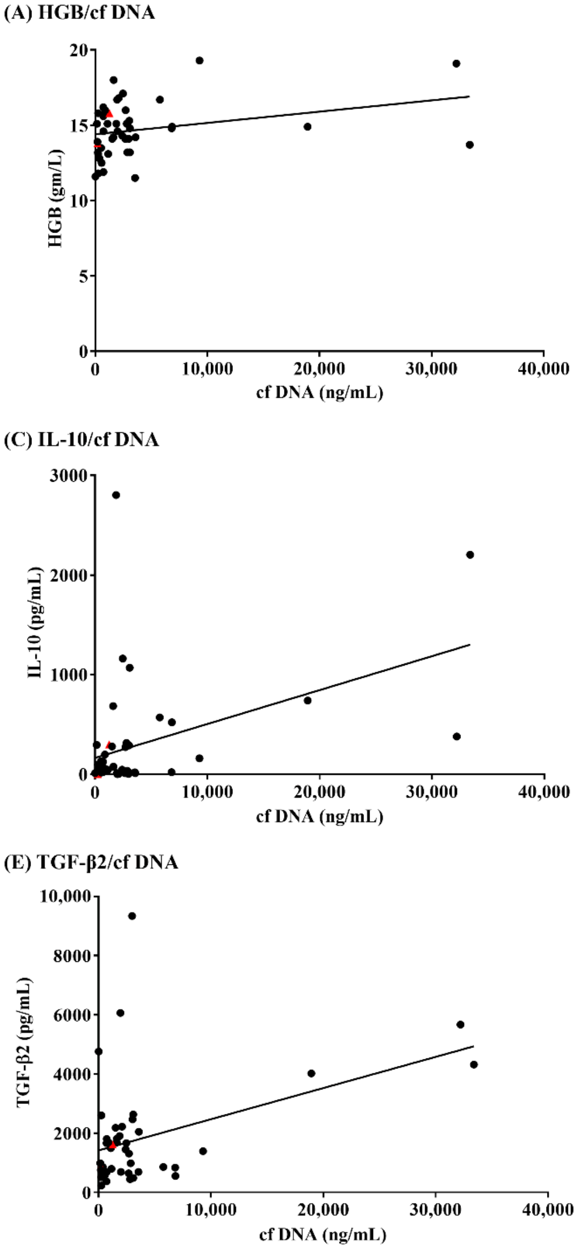

Figure S4. Correlation between biomarkers and cell-free DNA (cfDNA) in patients with hemorrhagic fever with renal syndrome. The serum concentrations of HGB (P = 0.0490), suPAR (P = 0.0082), IL-10 (P = 0.0031), CXCL10 (P = 0.0129), and TGF-β2 (P = 0.0043) were correlated to the cfDNA levels obtained from patients with HTNV infection. The correlation coefficient between the parameters is depicted by a straight line. Serum levels from patients who died (n=2) during Hantaan virus infection are shown in red triangles. Black circles indicate the measured 43 serum samples from 26 patients with HFRS. Abbreviations: HGB, hemoglobin.
